# Inverse Kinematics Solution of 6-DOF Manipulator Based on Multi-Objective Full-Parameter Optimization PSO Algorithm

**DOI:** 10.3389/fnbot.2022.791796

**Published:** 2022-03-17

**Authors:** Sha Luo, Dianming Chu, Qingdang Li, Yan He

**Affiliations:** College of Electromechanical Engineering, Qingdao University of Science and Technology, Qingdao, China

**Keywords:** inverse kinematics, multi-objective full-parameter optimization particle swarm optimization, inertia weight, asynchronous learning factor, time factor

## Abstract

A multi-objective full-parameter optimization particle swarm optimization (MOFOPSO) algorithm is proposed in this paper to overcome the drawbacks of poor accuracy, low efficiency, and instability of the existing algorithms in the inverse kinematics(IK) solution of the manipulator. In designing the multi-objective function, the proposed algorithm considers the factors such as position, posture, and joint. To improve PSO, the proposed algorithm comprehensively analyzes all factors affecting the global and local searching abilities. Based on this, the initial population is designed following the localized uniform distribution method. Meanwhile, the inertia weight, asynchronous learning factor, and time factor are respectively designed by introducing the iteration factor. Finally, this paper tests the performance of MOFOPSO with three typical functions to obtain a better inverse kinematics solution of the 6-DOF manipulator. Also, six other algorithms are taken for performance comparison. The experimental results indicate that the proposed method not only ensures the stability of the manipulator but also achieves high accuracy and efficiency in solving the inverse kinematics of the 6-DOF manipulator.

## Introduction

Recently, with the increase of the application scenarios and the number of mechanical arms, achieving high precision, and stability of the manipulator motion control system has attracted much attention. As an important factor to determine the motion precision and stability of the manipulator, kinematics modeling, and solving of manipulator has become a research hotspot. Functionally, kinematics can be divided into forward and inverse kinematics. With the change of each joint angle,the position and attitude of the end-effector of the manipulator can be obtained by using forward kinematics. This process represents the transformation from joint space to Cartesian space. The inverse kinematics solves the variation of the joint angles of the manipulator based on the position and attitude of the end-effector. Since the kinematics equation of a manipulator is a set of nonlinear equations, the solution to these equations must consider the existence of single solution and multi-solution and solution method. Compared to forwarding kinematics, inverse kinematics is more difficult to model and solve.

The study of inverse kinematics is crucial in the field of robotics research. This is not only because inverse kinematics is the basis of robot trajectory planning motion control and workspace analysis, but also it is a technical problem in robotics (Shi et al., 2020). With the continuous study of inverse kinematics, the inverse kinematics of the manipulator can be solved by four methods, including geometric method, analytical method, numerical method, and artificial intelligence method. The geometric method was first proposed to solve inverse kinematics. The paper (Xie et al., 2006) proposed a geometric method for the inverse kinematics of a 2-DOF manipulator. However, the process of solving the inverse kinematics of the multi-DOF manipulator is very complex, and the geometric method does not work. The most useful method for solving this problem is the analytical and numerical methods. Before solving inverse kinematics, the analytical method must analyze the number and structure of the joint of the manipulator. Craig (2009), Murray (2017), and Angeles and Angeles (2002) exploited the analytical method to solve the IK of the decoupled manipulator. In Funda and Paul (1990) concluded that quaternion is the most economical point transformation operator for the rigid screw displacement problem. In Raghavan and Roth (1993) proposed the elimination method as the solving method for the IK of the 6-DOF manipulator, in which the solution to each position equation of the manipulator was improved in the form of a six-member quadratic polynomial. In Husty et al. (2007) combined double quaternion with Segre flow patterns for the IK of the 6-DOF manipulator. Although the analytical method is very efficient in solving the IK equation, there are some shortcomings such as low accuracy, poor real-time performance, and difficulty in ensuring stable operations of the manipulator in a dynamic environment. In contrast, there are a variety of numerical methods that can be exploited to solve inverse kinematics, such as Newton's method, Jacobian iterative method (Buss, 2004), and mixed inverse kinematics method. Because the Jacobian iteration method has a simple principle and no special requirements on the structure and number of joints of the manipulator, it is exploited as a method for solving most IK problems. Meanwhile, the numerical method also has the advantage that it can obtain an inverse solution containing path planning from the initial pose to the target pose, so it is suitable for path planning. However, in theory, it is impossible to verify whether the inverse solution is optimal or suboptimal.

With the continuous development of artificial intelligence and intelligent algorithms, some artificial intelligence methods have been used to solve the inverse kinematics of manipulators and have achieved good effects. And compared with geometric, numerical and analytical methods to solve the inverse kinematics of manipulator, the artificial intelligence algorithm does not need to carry out derivation operation to obtain jacobian matrix, and does not have the singularity problem in the conventional iterative method (Shi et al., 2020). And artificial intelligence algorithm has no special requirements for robot mechanism and has strong universality,so it is applied to parallel (Kucuk, 2012) and hybrid robots (Tanev, 2000; Serdar and Baris Doruk, 2016). The most widely used algorithms include ANN(artificial neural network), GA(genetic algorithm), and PSO. Hasan et al. (2006) proposed an ANN-based adaptive learning algorithm to overcome the uncertainty and nonlinearity in solving IK equations. Momani et al. (2016) solved the IK of the manipulator with GA, which obtained path planning in the iterative process. Ma et al. (2016) proposed to use ANN to solve the IK of the manipulator. Khaleel (2018) proposed a combination of neural network algorithm and GA for the IK of the manipulator. Although ANN and GA have achieved good results in solving the IK problem, both of them have some defects. ANN suffers from a long training time and weak generalization ability, while GA has a long iteration time and easily falls into local optimum. In 2018, Nizar and Alimi (2013) used PSO and improved PSO to solve the IK problem, and they compared the simulation results of the two algorithms. In 2013, Idris et al. (2018) proposed an adaptive PSO. They applied this algorithm to solve the IK of a 3-DOF manipulator and achieved good results. Dereli and Koker (2018) proposed an adaptive weighted PSO (AWPSO) algorithm to achieve high position accuracy. The 7-DOF manipulator was taken as the object for simulation experiments, and the simulation results were compared with those of the traditional PSO algorithm.

All the above studies only focused on solving the IK problem of the manipulator but rarely conducted a comprehensive and in-depth study of the IK of the manipulator. However, the development of the IK algorithm is limited by the accuracy of position and attitude and the stability of the manipulator. Aiming at the poor accuracy and low efficiency in solving the manipulator and the instability in running the manipulator, the MOFOPSO algorithm is proposed in this paper. Firstly, the algorithm minimizes the manipulator's position error, attitude error, and joint angle change. Then, it transforms the solution of the inverse kinematics of the manipulator into a multi-objective optimization problem.

## Kinematics Analysis of Manipulator

**Figure 1:**
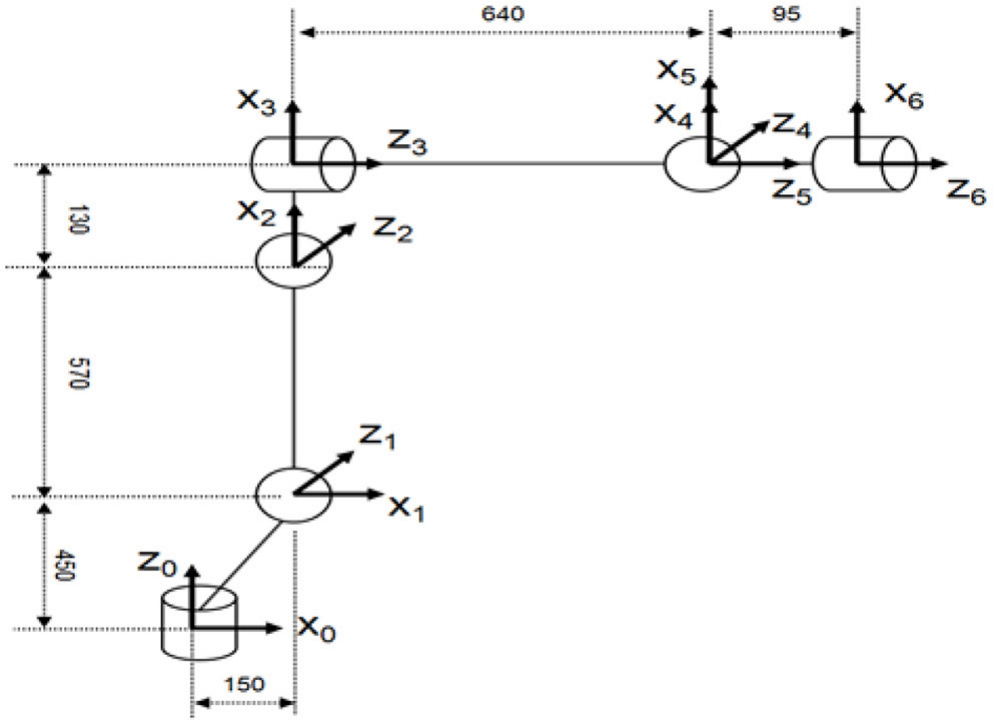
The coordinate system of the 6-DOF manipulator.

For further studying the kinematics of the manipulator, this paper seper selects the 6-DOF manipulator as the research object. Firstly, the kinematic model of the manipulator needs to be established. In terms of kinematic model, literature (Ayiz and Kucuk, 2009) uses exponential rotation matrix to directly describe the physical mechanism of the manipulator, but the multiple transformations of matrix produce a large amount of calculation.In this paper the coordinate system of the 6-DOF manipulator is established according to the Danavit and Hartenberg model (Danavit and Hartenberg, 1964), which is shown in Figure 1.

In terms of the adjacent coordinate systems of the manipulator*i*and *i*−1, the homogeneous coordinate transformation matrix ${}_{i}^{i-1}T$ can be used to express the relative relation of the position and pose.


(1) $${}_{i}^{i-1}T=\left[\begin{array}{cccc} \cos\theta_{i} & -\sin\theta_{i}\cos\alpha_{i} & \sin\theta_{i}\sin\alpha_{i} & a_{i}\cos\theta_{i}\\ \sin\theta_{i} & \cos\theta_{i}\cos\alpha_{i} & -\cos\theta_{i}\sin\alpha_{i} & a_{i}\sin\theta_{i}\\ 0 & \sin\alpha_{i} & \cos\alpha_{i} & d_{i}\\ 0 & 0 & 0 & 1 \end{array}\right]$$

According to the DH model, it is easy to obtain the model of the 6-DOF manipulator's forward kinematics, and the model can be expressed as follows:


(2) $${}_{6}^{0}T{=}_{1}^{0}T_{2}^{1}T_{3}^{2}T_{4}^{3}T_{5}^{4}T_{6}^{5}T=\left[\begin{array}{cccc} n_{x} & o_{x} & a_{x} & p_{x}\\ n_{y} & o_{y} & a_{y} & p_{y}\\ n_{z} & o_{z} & a_{z} & p_{z}\\ 0 & 0 & 0 & 1 \end{array}\right]$$

Where ${}_{6}^{0}T$ represents the position matrix of the end-effector of the mechanical arm relative to the inertial coordinate system; ${}_{i}^{i-1}T$ represents the homogeneous transformation matrix of adjacent coordinate systems.

The IK of the manipulator can be solved by obtaining the pose matrix of the end-effector of the manipulator and solving the changes of the joint angles of the manipulator. The DH parameters of the 6-DOF manipulator used in this paper are listed in Table 1.

**Table 1:** The DH parameters of the 6-DOF manipulator.

***i***	***d*_*i*_(*mm*)**	**α_*i*_(*Deg*)**	***a*_*i*_(*mm*)**	**θ_*i*_(*Deg*)**	**θ_*i*_(*Degree*)**
1	450	−90	150	θ_1_	−170~170
2	0	0	570	θ_2_	−110~155
3	0	−90	130	θ_3_	−80~210
4	640	90	0	θ_4_	−190~190
5	0	−90	0	θ_5_	−50~230
6	95	0	0	θ_6_	−360~360

Based on the parameters in the DH table and equations (1) and (2), the expression of the pose matrix of the end-effector of the manipulator can be obtained.

## The Proposed Multi-Objective Full-Parameter Optimization Particle Swarm Optimization Method

### Classical PSO Method

With the progress of the research on the IK of manipulators, artificial intelligence has been exploited to solve the inverse kinematics, and the swarm optimization algorithm has been widely used in this field. As early as 1995, Eberhart and Kennedy (1995) proposed PSO under the inspiration of birds' foraging behavior. PSO simulates the birds in the flock in the form of massless particles and gives two attributes of the particle, i.e., the speed and direction of movement. During the whole search process, there are two extreme values. One is the current individual extreme value,which is searched by the individual particle separately. Also, the individual particle exchanges information with other particles in the group. Another extreme value is the group extreme value, which is updated into the best individual extreme value. Based on the two extreme values, all particles in the group dynamically adjust their velocity and position,and the update equations are as follows:

(3) $$\begin{array}{r} v_{jd}\;=\;v_{jd}\;+\;c_{1}r_{1}\left(p_{best}\;-\;x_{jd}\right)\;+\;c_{2}r_{2}\left(g_{best}-x_{jd}\right) \end{array}$$

(4) $$\begin{array}{r} x_{jd}\;=\;x_{jd}\;+\;v_{jd} \end{array}$$

Where, *X*_*j*_ = (*x*_*j*1_, *x*_*j*2_, …*x*_*jD*_) is the position of the *j* particle; *P*_*j*_ = (*p*_*j*1_, *p*_*j*2_, …*p*_*jD*_)denotes the extreme value; *g*_*best*_ is the population extreme value; *v*_*jd*_ is the velocity of the particle; *c*_1_ and *c*_2_ are constants representing the learning coefficient; *r*_1_ and *r*_2_ are random real numbers in the range of [0, 1]; *d* = 1, 2, 3⋯ , *D* is the dimension, and *j* = 1, 2, 3, …*S* is the *j* particle.

**Figure 2:**
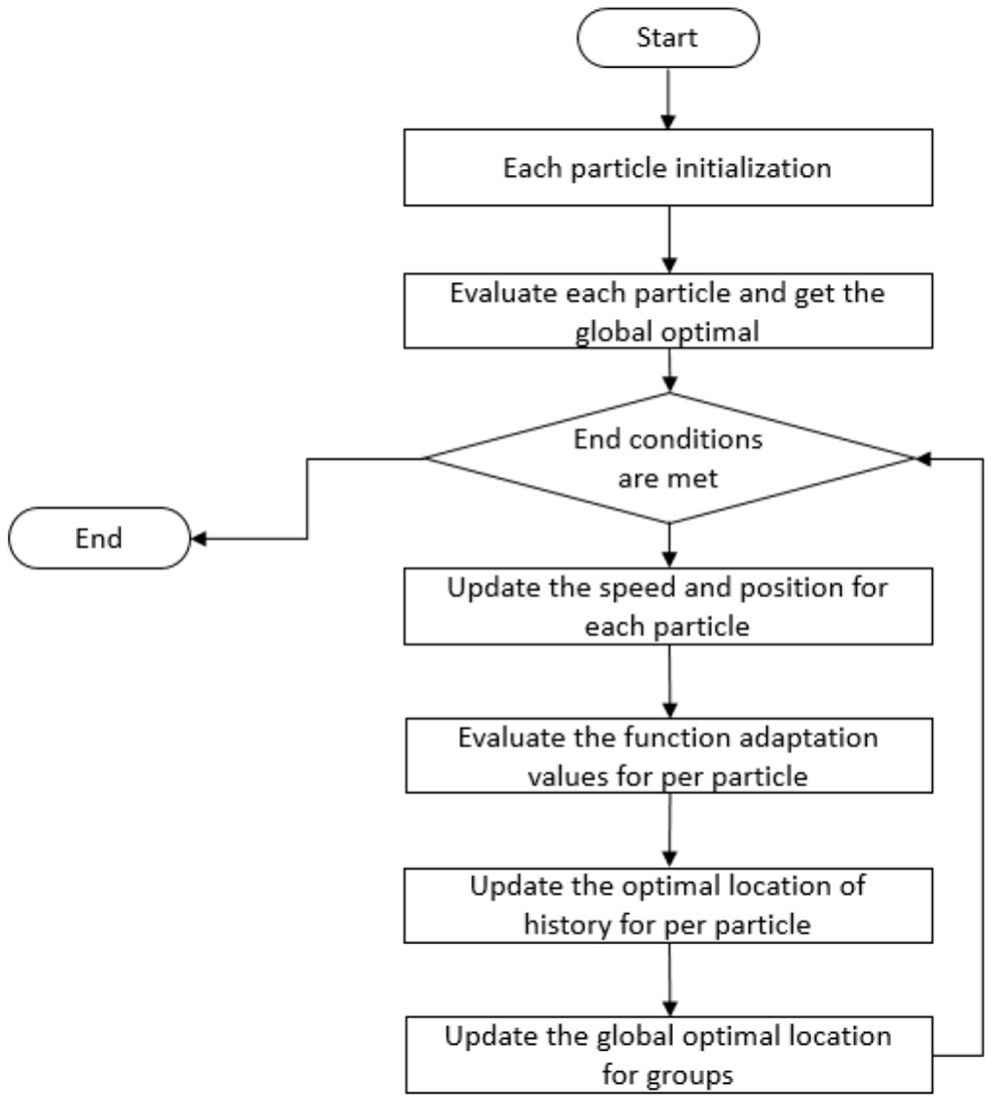
The flow chart of the traditional PSO used as the IK solution of the manipulator.

The flow chart of using the classical PSO to solve the IK of the manipulator is shown in Figure 2.

### Multi-Objective Full-Parameter Optimization PSO Method

In the intelligent algorithm, the PSO algorithm has many advantages, such as easy implementation, fewer tuning parameters, and fast convergence,so it can obtain good results in the application field. However, in many cases, the classical PSO algorithm is easy to fall into the local optimum. Therefore, the studies (Shi and Eberhart, 1998a,b) optimize the traditional PSO algorithm for balancing global and local search capabilities by changing the inertia weight. Besides the inertia weight, the searching ability and efficiency of the PSO algorithm are affected by the selection of learning factors, initial population, and fitness function. In recent years, researchers have improved various particle swarm optimization algorithms in Table 2. So, considering these factors, the initial population is designed in this paper following the localized uniform distribution method. Then, the inertia weight, asynchronous learning factor, and time factor are respectively designed following the method of introducing the iteration factor. Finally, a multi-objective PSO algorithm with full parameters is designed in combination with the multi-objective optimization algorithm. The proposed algorithm makes the particle swarm in the initial state more representative and obtains the optimal solution quickly. Also, it integrates the inertia weight and learning factor into the design of the factors and the process of location update algorithm to balance global and local search capabilities, thus improving the diversity and generalization ability of the algorithm.

**Table 2:** Particle swarm optimization and its improved algorithm.

**References**	**Algorithm**	**Improve**
Eberhart and Kennedy, 1995	PSO	Based on the flock-based predation behavior
Lovbjerg et al., 2001	Breed	With the advantages of both two algorithms, faster speed and higher accuracy
Zhan et al., 2009	Adaption	Inertial weights change and converge faster than the Breed Algorithm
Lili et al. (2015)	Lin	Improve the ability to find excellence, reduce the oscillation phenomenon
Yaming (2019)	Nature	More optimization ability and solution speed, reduce precocious convergence

#### Initialize the Population Design

The selection of the initial population directly affects the search efficiency of the PSO algorithm. Theoretically, the optimal solution of the problem is unknown at the initial stage of the algorithm. So, if the initial population is randomly generated, the population representation is poor, which can affect the efficiency of particle swarm search. Meanwhile, with the increase in the dimensionality of the search space, the algorithm is easy to fall into local optimum because of the randomness of the initial position of particle swarm optimization. If the particles can be uniformly distributed in the feasible region during initialization, the efficiency of the global search can be guaranteed, and the probability of searching for the optimal solution by the population can be improved. In response to this problem, a limited domain uniform distribution method is proposed to generate the initial population. Considering the robot joint angle restriction, the initial value of each dimensional particle swarm is uniformly distributed within this range.

#### Optimal Inertia Weight Strategy

In PSO algorithms, inertia weight is a very important parameter to balance the global and local search capabilities of the whole algorithm. If the inertial weight is too large, the global search capability is strong but the local search capability is poor; if the inertial weight is too small, the local search capability is strong but the global search capability is poor (Xi-Hu, 2011). Although the decline of linear weight can improve the balance of the global and local search capabilities of the algorithm to some extent, the local search capability will be weakened as the number of iterations decreases linearly. It is found that a large inertial weight is conducive to the global search at the beginning of the iteration, while a small inertial weight is conducive to local precise search at the later stage of the iteration. However, a fast decline process is easy to cause the problem of search fault. Therefore, this paper designed the nonlinear function of optimal inertia weight by introducing the factor of iteration number. Based on this, the range can be quickly determined at the beginning of the iteration. Then, the local precise positioning is realized with a small change of inertia weight to obtain the optimal inertia weight at a fast speed. The function is as follows:

(5) $$\begin{array}{r} w\;=\;w_{max}-\left(w_{max}-w_{min}\right)sin\left(\frac{r\pi }{2N}\right) \end{array}$$

Where,*w*∈[*w*_min_, *w*_max_]; *r* and *N* are respectively the current iteration and total iterations number of the algorithm.

#### Asynchronous Learning Factor

The learning factor is usually set to a constant in the classical PSO algorithm. But it is an important factor that affects the self-cognition and social cognition of particles and the movement direction and position of particles. Also, it reflects the degree of information communication among particles in the whole population. To make the global search ability better, at the beginning of the algorithm iteration, the particles are expected to have more group communication, that is, strong social cognition ability, which facilitates the global search. In later iterations, the self-cognition ability of the particle is expected to be strong to achieve an accurate local search. Based on the analysis, the function and iteration number factor are introduced into the learning factor design in this paper. Also, in the early iteration, the learning factor is adjusted to achieve a strong social cognition ability and a strong self-cognition ability in the late iteration, Besides, the optimal learning factor is quickly realized through nonlinear changes. The learning factors are set as follows:


(6) $$\{\begin{array}{c} c_{1}=c_{min}+(c_{max}-c_{min})\frac{2}{1+e^{-(1{+}^{20r}\text{​​}╱\text{​}{\text{​}}_{N}\;)}}\\ c_{2}=c_{max}-(c_{max}-c_{min})\frac{2}{1+e^{-(1{+}^{20r}\text{​​}╱\text{​}{\text{​}}_{N}\;)}}\; \end{array}$$

Where, *c*_1_, *c*_2_∈[*c*_min_, *c*_max_]; *r* and *N* are respectively the current iteration and total iterations number of the algorithm.

#### Time Factor

The position update strategy of the classical PSO is based on the initial position and the current speed. From the perspective of physics, the addition of two physical quantities requires the quantities have the same dimension, which means that the direct addition of displacement and velocity does not conform to the physical theory. Therefore, in the classical PSO algorithm, the position update formula contains a time factor that is important for particles to oscillate near the optimal solution. However, this time factor is usually set to 1 (Kong et al., 2010). To improve the particle search ability, the time factor is adopted by this paper. In equation (4), a time factor is added to the local update model, and the update function is as follows:

(7) $$\begin{array}{r} x_{id}=x_{id}+Tv_{id} \end{array}$$

Through experiments, it is found that the convergence of the algorithm is affected by the time factor. So, the iteration number is introduced to design the time factor:

(8) $$\begin{array}{r} T=0.5+\frac{r}{2N} \end{array}$$

Where, *r* and *N* are respectively the current iteration and total iterations number of the algorithm.

#### Multi-Objective Function

Multi-objective optimization (He and Shao-hua, 2019) is complex, and it is widely used in engineering applications. However, in this optimization problem, each objective cannot achieve the optimal at the same time due to non-linearity, multi-dimension, and other characteristics. Also, each objective must have a weight. In this case, the allocation of the weight becomes a hot research topic. There are a variety of traditional multi-objective optimization methods, such as the weighted summation method (Zadeh, 1963), objective programming method (Charnes et al., 1955), maximum and minimum value (Tseng and Lu, 1990), etc. The key of these methods is to transform the multi-objective problem into a single-objective problem and then use the single objective algorithm for optimization. However, these methods rely too much on experience and fail to achieve the optimization effect when the multi-objective problem has non-linearity and high latitude. Recently, with the continuous development of the evolutionary algorithm along with its unique update mechanism, various evolutionary algorithms have been applied to the combinatorial optimization and numerical optimization fields (Liu et al., 2020) and achieved breakthroughs. The MOPSO algorithm (Li et al., 2017) is one of the typical multi-objective algorithms. Due to the precocity of particle swarm in the traditional multi-objective PSO algorithm, the particle swarm will suffer from local optimization, poor convergence, global search, and local search ability imbalance (Feng et al., 2020). In this section, the multi-objective function will be optimized and improved for the IK of the manipulator to make the MOPSO algorithm have excellent optimization performance and pertinence.

Based on the multi-objective optimization and taking the 6-DOF manipulator as the object, this paper designs a multi-objective function to minimize position error, attitude error, and joint angle change:


(9) $$\{\begin{array}{ll} F & =Min(f_{1}(p_{tar}),f_{2}(e_{tar}),f_{3}(\theta_{i}))\\ \; & s.t.\theta_{i\min}\leq \theta_{i}\leq \theta_{i\max}\; \end{array}$$

Where, *F* is the multi-objective function; *f*_1_(*p*_*tar*_), *f*_2_(*e*_*tar*_), and *f*_3_(θ_*i*_) are respectively the position error function, the attitude error function, and the joint angle change function; θ_*i*min_ and θ_*i*max_ are the ranges of joint angle θ_*i*_.

To make the algorithm more targeted, the structural principle of the manipulator is studied in-depth, and then the position error function, attitude error function, and angle change function of each joint are designed. Especially, the joint angle change function involves the changes of multiple joint angles. If it is designed with the minimum changes of all joint angles, it does not conform to the structural principle of the manipulator. Specifically, there is the least change in the inertial coordinate system of the manipulator in the whole operation process, while the joint changes at the end of the manipulator are generally large. So, the linear weighted summation method is adopted by this paper to design the joint angle change function. The mathematical description of the position error function, attitude error function, and joint angle change function is as follows:


(10) $$\{\begin{array}{ll} f_{1}(p) & =\left\|p_{tar}-H_{position}(q_{tar})\right\|\\ f_{2}(p) & =\left\|e_{tar}-H_{orientation}(q_{tar})\right\|\\ f_{3}(\theta_{i}) & =\sum_{i=1}^{6}{\text{λ}}_{i}\sqrt{{\left(\theta_{i}-\theta_{init}\right)}^{2}}\\ \sum_{i=1}^{6}{\text{λ}}_{i} & =1\\ i=1,2 & \cdots 6 \end{array}$$

Where, *p*_*tar*_ and *e*_*tar*_ are respectively the target position and attitude of the end-effector of the manipulator; *q*_*tar*_ is the angle value of each joint angle of the manipulator, and it can be solved by the equation; θ_*init*_ contains the initial values of the joint angles of the manipulator, and λ_*i*_ is the weight value of the change of joint angle θ_*i*_.

#### Fitness Function

The construction of the fitness function is the most important step in the PSO algorithm. For the inverse kinematics, this paper designs the fitness function by considering the position, attitude accuracy, and joint angle change at the same time. According to equation (9), the fitness function with the minimum variation of position accuracy, posture accuracy, and joint angle can be obtained:


(11) $$\{\begin{array}{ll} f_{1}(q_{cur}) & =\sqrt{{\left(p_{x_{-}tar}-p_{x_{-}cur}\right)}^{2}+{\left(p_{y_{-}tar}-p_{y_{-}cur}\right)}^{2}+{\left(p_{z_{-}tar}-p_{z_{-}cur}\right)}^{2}}\\ f_{2}(q_{cur}) & =\sqrt{{\left(e_{x_{-}tar}-e_{x_{-}cur}\right)}^{2}+{\left(e_{y_{-}tar}-e_{y_{-}cur}\right)}^{2}+{\left(e_{z_{-}tar}-e_{z_{-}cur}\right)}^{2}}\\ f_{3}(\theta_{i}) & =\sum_{i=1}^{6}{\text{λ}}_{i}\sqrt{{\left(\theta_{i}-\theta_{init}\right)}^{2}} \end{array}$$

Where,$p_{tar}={\left[p_{x\text{\_}tar},p_{y\text{\_}tar},p_{z\text{\_}tar}\right]}^{T}$ and *e*_*tar*_ = [*e*_*x*_*tar*_, *e*_*y*_*tar*_, *e*_*z*_*tar*_] are respectively the target position and attitude of the manipulator; $p_{cur}={\left[p_{x\text{\_}cur},p_{y\text{\_}cur},p_{z\text{\_}cur}\right]}^{T}$ and *e*_*cur*_ = [*e*_*x*_*cur*_, *e*_*y*_*cur*_, *e*_*z*_*cur*_] respectively represent the initial position and attitude of the manipulator; λ_*i*_ is the weight value of the change of joint angleθ_*i*_.

### IK Model of the 6-DOF Manipulator Based on MOFOPSO

Based on the MOFOPSO algorithm, the IK model of the 6-DOF manipulator can be obtained as follows:


(12) $$\{\begin{array}{ll} F & =Min(f_{1}(p_{tar}),f_{2}(e_{tar}),f_{3}(\theta_{i}))\\ \; & s.t.\theta_{i\min}\leq \theta_{i}\leq \theta_{i\max}\\ \; & v_{jd}=w\;\cdot \;v_{jd}+c_{1}(p_{best}-x_{jd})+c_{2}(g_{best}-x_{jd})\\ \; & x_{jd}=x_{jd}+Tv_{jd}\\ w & =w=w_{max}-(w_{max}-w_{min})sin\left(\frac{r\pi }{2N}\right)\\ c_{1} & =c_{min}+(c_{max}-c_{min})\frac{2}{1+e^{-(1{+}^{20r}\text{​​}╱\text{​}{\text{​}}_{N}\;)}}\\ c_{2} & =c_{max}-(c_{max}-c_{min})\frac{2}{1+e^{-(1{+}^{20r}\text{​​}╱\text{​}{\text{​}}_{N}\;)}}\;\\ \;T & =0.5+\frac{r}{2N}\\ f_{1}(p) & =\left\|p_{tar}-H_{position}(q_{tar})\right\|\\ f_{2}(p) & =\left\|e_{tar}-H_{orientation}(q_{tar})\right\|\\ f_{3}(\theta_{i}) & =\sum_{i=1}^{6}{\text{λ}}_{i}\sqrt{{\left(\theta_{i}-\theta_{init}\right)}^{2}}\\ \; & \sum_{i=1}^{6}{\text{λ}}_{i}=1\\ i & =1,2\cdots 6\; \end{array}$$

Where all the variables in the formula have been introduced in the above description.Among them, the value of λ_i_ (*i* = 1,2..6) is decreasing, i.e., λ_1_>λ_2_ >λ_3_>λ_4_ >λ_5_ >λ_6_. The linear decline method is adopted in this calculation.

The proposed MOFOPSO algorithm analyzes the structure characteristics of the mechanical arm and the instability problem in the process of mechanical arm movement. Also, it analyzes the defects of traditional inverse kinematics algorithms, such as only considering location accuracy or precision of position and attitude. So, the proposed algorithm is a combination of theory and application and has certain practicability.

## Algorithm Performance Test

In this paper, six algorithms are taken for performance test, including the classical PSO algorithm, random weight PSO (RWPSO) (Zhan et al., 2009), linear decline PSO (LDPSO) (Lili et al., 2015), PSO algorithm based on hybridization (Lovbjerg et al., 2001), PSO algorithm based on natural selection (Yaming, 2019), and PSO algorithm based on simulated annealing. However, in the performance evaluation process, the PSO algorithms based on simulated annealing and natural selection achieve poor performance in convergence quality, speed, and stability. Thus, they are excluded from the evaluation of test function, convergence speed, and stability, while the performance of the rest algorithms is compared.

Although many articles (Yiyang et al., 2021) use many test functions to test the performance of PSO algorithm, but in this paper, MOFOPSO algorithm is proposed to overcome the drawbacks of poor accuracy, low efficiency. SO three test functions are chosen for the algorithm performance test, including Ackely function, Rastrigin function, and Griewangk function to investigate the shortcomings of classical PSO algorithms, such as slow convergence speed and falling into local optimum.

### Convergence Rate Test

**Figure 3:**
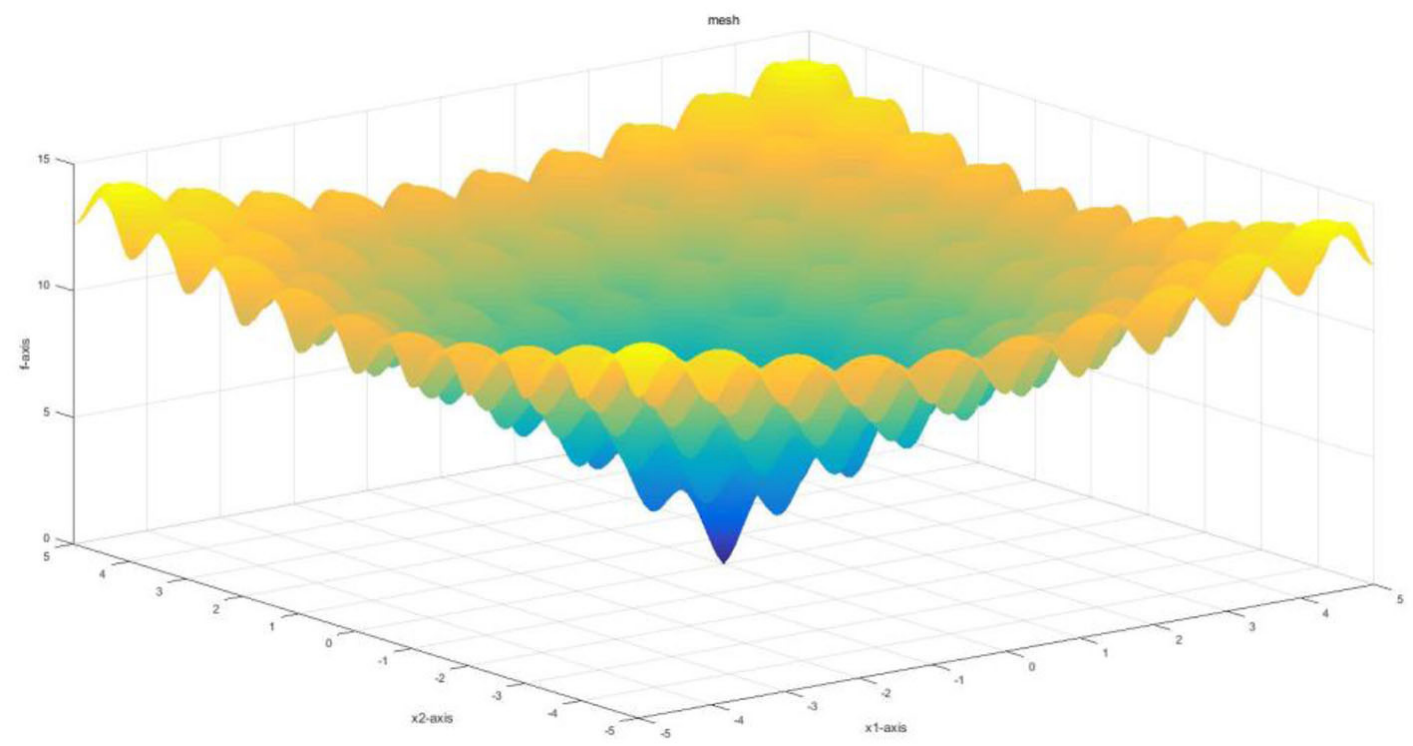
The image of the Ackely function.

Ackely function is a continuous function used to evaluate the convergence rate of an algorithm. It is obtained by superposing the exponential function with a moderately amplified cosine. As shown in Figure 3, the Ackely function presents the multi-directionality of a multi-dimensional point in the optimization process. Thus, the Ackely function is used to detect the global convergence rate of the algorithm.

**Figure 4:**
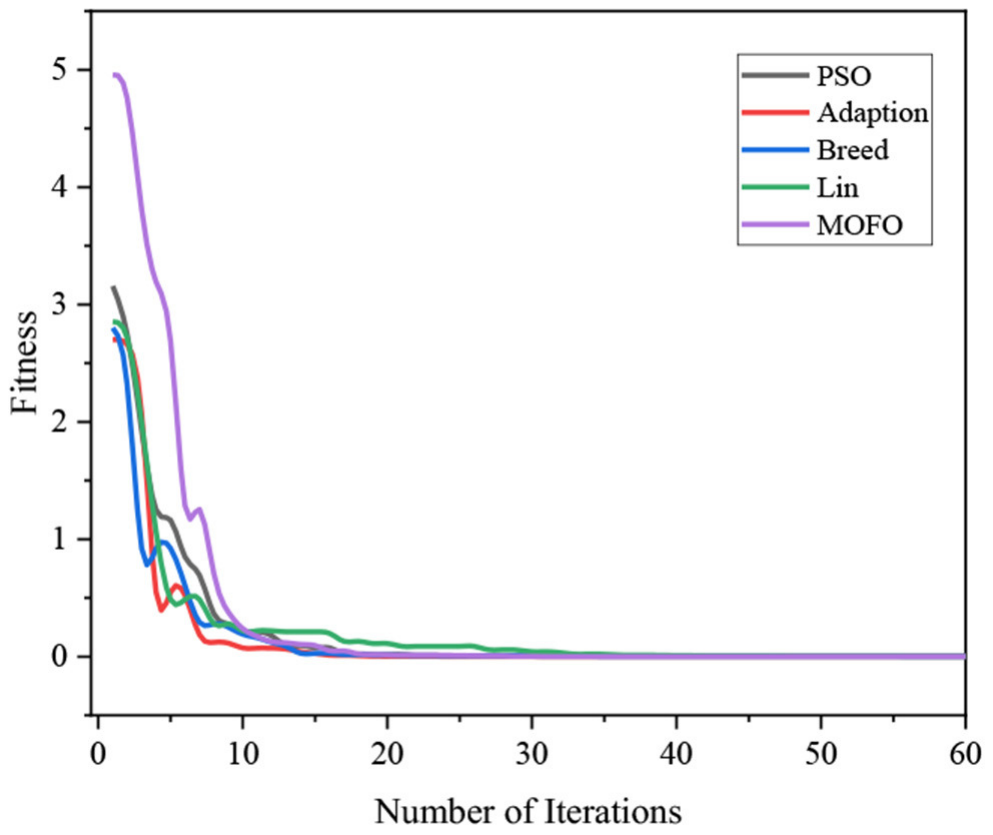
The comparison of the convergence speed of different algorithms.

Figure 4 demonstrates the results obtained by the six algorithms using the Ackely function. It indicates that the RWPSO and MOFOPSO algorithms achieve the fastest convergence speed, while the MOFOPSO is better than the RWPSO at a close speed. From the vertical axis of the image, it can be seen that the fitness function value of the MOFOPSO algorithm rapidly decreases from the large value to the optimal value. This meets the design goal of balancing the global and local search capabilities of the algorithm.

### Convergence Test

**Figure 5:**
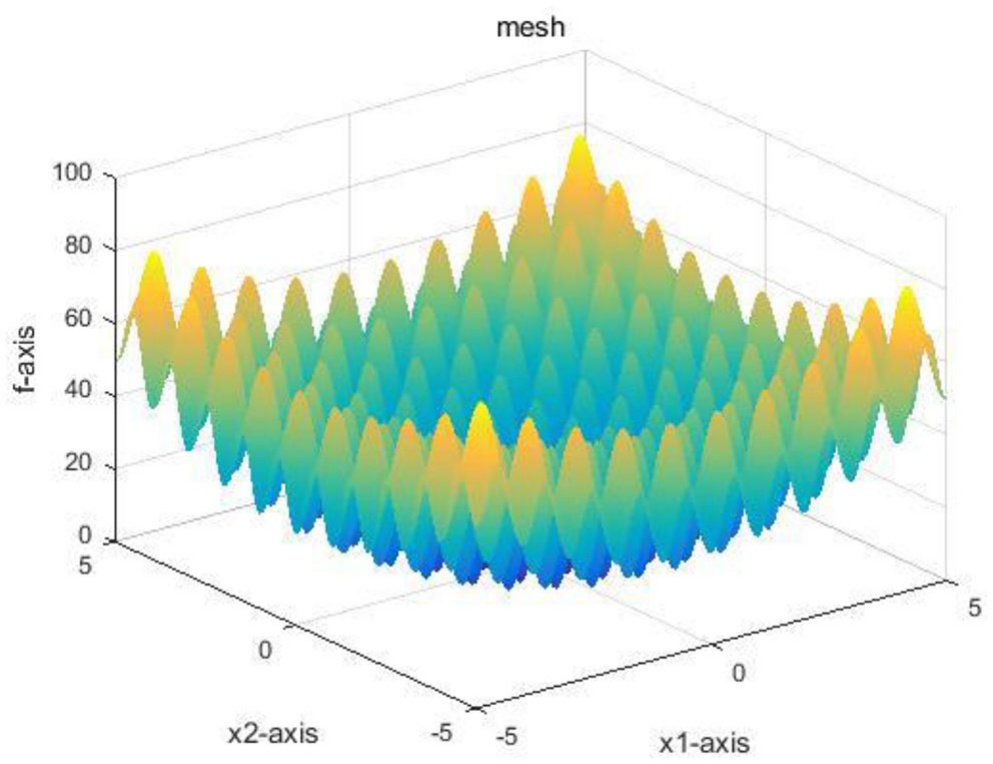
The image of the Rastrigin function.

The Rastrigin function is a highly multimodal function used to test the ability of global optimization. This function is based on the De Jong function and adds a cosine modulation transfer function to generate frequent local minima. This paper makes good use of the Rastrigin function to test the practicability of the algorithm for a case with regular solutions. The figure of the Rastrigin function is illustrated in Figure 5.

**Figure 6:**
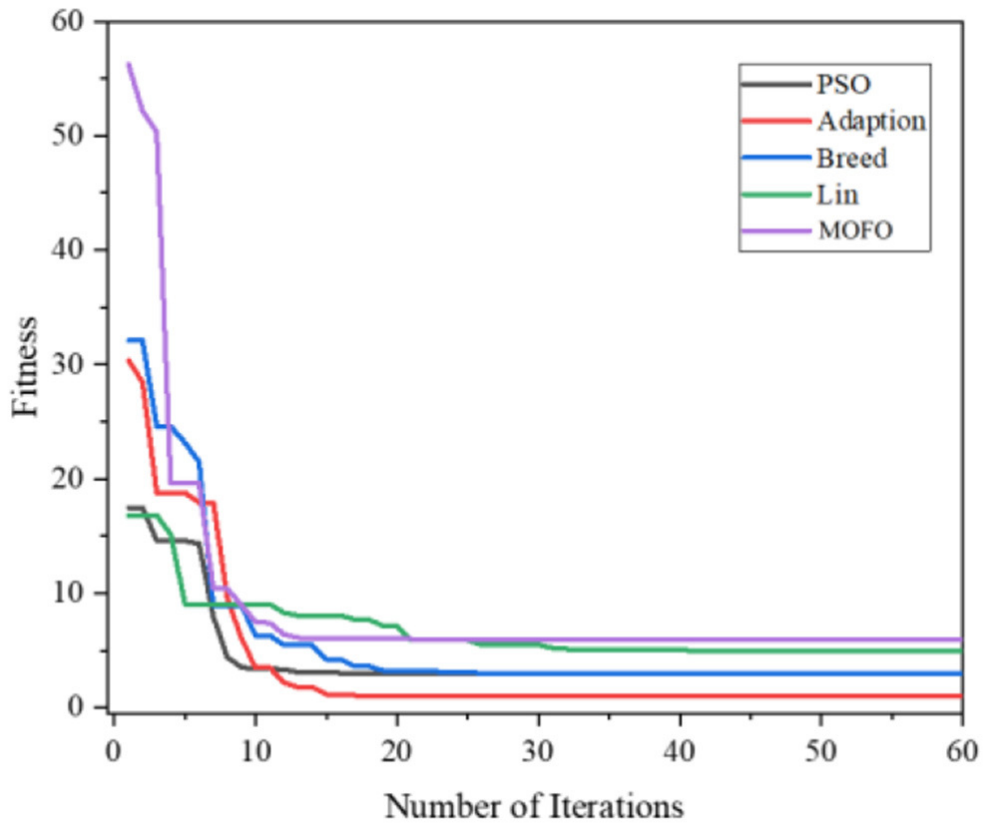
The Comparison of the convergence speed of different algorithms.

Figure 6 demonstrates the convergence of different algorithms. It can be seen that all the algorithms converge well, and the proposed MOFOPSO algorithm converges faster than the other algorithms with no shocks.

### Skip the Local Optimal Capability Test

**Figure 7:**
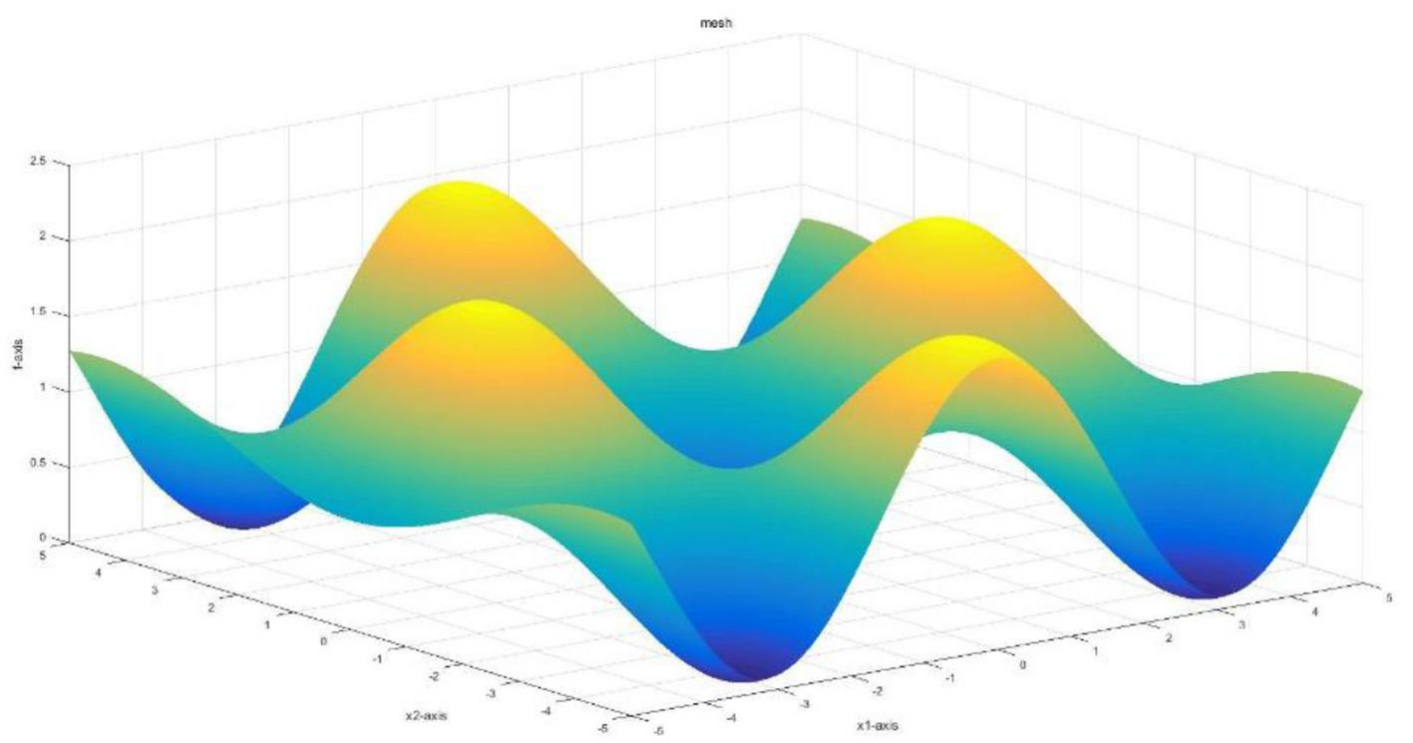
The image of the Griewangk function.

The Griewangk function is used to detect the ability to jump out of the local optimum. The function changes with the quantity, and there are a large number of local extreme values in the real data distribution of the function. The image of the Griewangk function is illustrated in Figure 7. This function can be exploited to detect the convergence of the algorithm with a regular position of minimum value.

**Figure 8:**
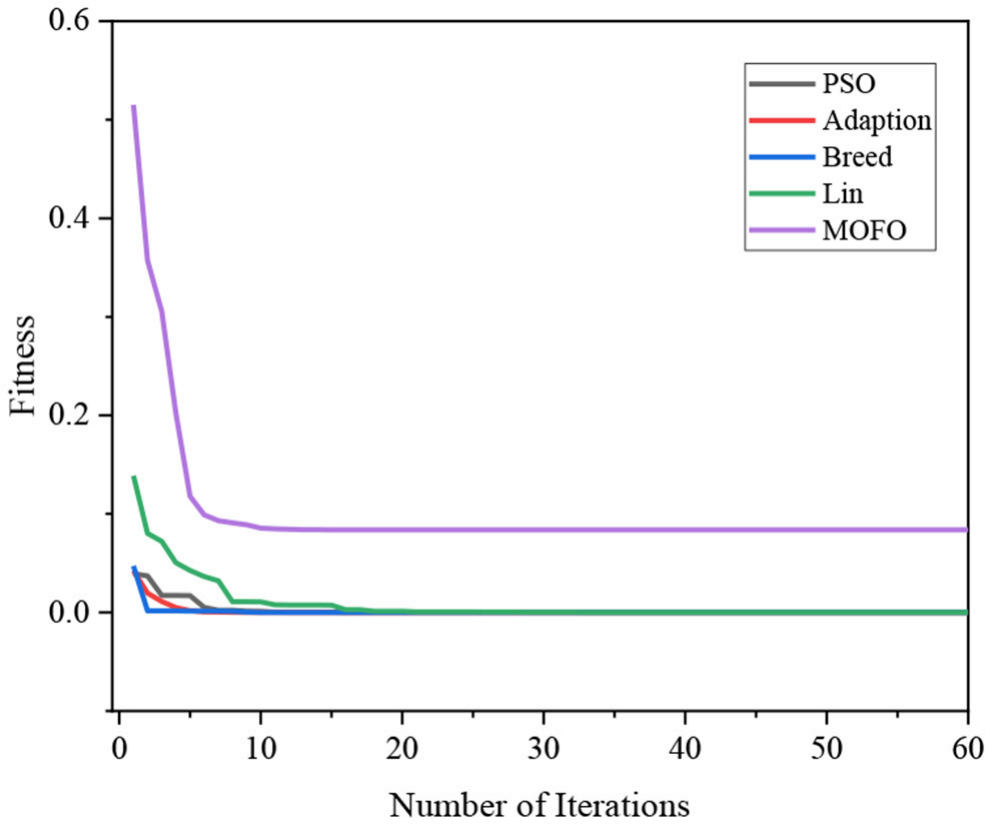
The abilities of the algorithms to jump out of the local optimal.

Figure 8 demonstrates the abilities of the algorithms to escape from the local optimum. It can be seen that the multi-objective full-parameter optimization particle swarm optimization algorithm can escape from the local optimum at a faster speed than other algorithms.

Above all, the three test functions, Ackely function, Rastrigin function, and Griewangk function, are all multi-modal functions, which are considered to be complex functions that are difficult for the optimization algorithm to solve. However, the proposed MOFOPSO algorithm can solve the complex multi-modal function optimization difficulty and performs better in terms of convergence speed, convergence, and the ability to escape from the local minimum value than other algorithms. And it should be noted that in the figure comparing the test functions (Figures 6–8), it can be seen that the initial value is the fitness function value. Due to our fitness function on the traditional PSO algorithm was improved, the mechanical arm joint Angle change values as a fitness function of a component, and the change of the mechanical arm joint Angle value compared to the position and posture error value is bigger, which makes the whole parameter optimization PSO to calculate the fitness function value is higher than other fitness function value of the algorithm.

Above all, we can obtain the initial and convergent values of different algorithms in Table 3. Although the initial value of the MOFOPSO algorithm is bigger than others, the gradient of the MOFOPSO algorithm shows that this algorithm has the fastest convergence speed.

**Table 3:** The initial and convergent values of different algorithms.

	**Algorithm**	**Initial value**	**Convergent value**	**Iterative step**	**Gradient**
Ackely	PSO	3.156917	0.017917	19	0.165211
	Adaption	2.699713	0.013031	16	0.167918
	Breed	2.800934	0.017496	17	0.163732
	Lin	2.852798	0.129628	17	0.160186
	MOFO	4.957359	0.050007	16	0.30671
Rastrigin	PSO	0.03943727	0.002264	7	0.0053105
	Adaption	0.04220217	0.000696	6	0.0069178
	Breed	0.04718385	0.001574	2	0.0228052
	Lin	0.13877033	0.007731	11	0.0119127
	MOFO	0.51498297	8.55E55E-02	10	0.0429484
Griewangk	PSO	17.48753	3.39924	10	1.408829
	Adaption	30.34479	3.49541	10	2.684937
	Breed	32.09457	8.848614	7	3.320851
	Lin	16.80333	9.048955	5	1.550875
	MOFO	56.26652	10.38	7	6.555218

## Results Discuss

Based on the algorithm performance test, a 6-DOF manipulator from Japan Yaskawa Company is taken to simulate the solution to the inverse kinematics problem. Meanwhile, six algorithms are selected for performance comparison, and the simulation results of the MOFOPSO algorithm proposed in the article are analyzed to further verify the superiority of the performance of the algorithm.

### Parameter Setting Analysis

When the MOFOPSO algorithm is simulated, it is not necessary to set the relevant parameters of the algorithm but to input the parameters of the 6-DOF manipulator. However, for other algorithms,it is necessary to set the relevant parameters (inertia weight, learning factor, or one of both) to the empirical values during simulation. Thus, in terms of parameter setting, the MOFOPSO is more practical, effective, and intelligent than other algorithms. The relevant parameters of various algorithms are listed in Table 4.

**Table 4:** The relevant parameters of various algorithms.

**PSO variants**	**Parameters**
PSO	c_1_=2,c_2_=2,w=0.5
PSO_Adaptation	c_1_=2,c_2_=2,w_max_=0.8,w_min_=0.2
PSO_Breed	c_1_=2,c_2_=2,w=0.5,b_c_=0.8,b_s_=0.05
PSO_Lamda	c_1_=2,c_2_=2,lamda=0.99
PSO_Lin	c_1_=2,c_2_=2,w_max_=0.8,w_min_=0.2
PSO_Nature	c_1_=2,c_2_=2,w=0.5
MOFOPSO	C_max_=3,c_min_=1.5, w_max_=0.8,w_min_=0.2 (c_1_=2.99,c_2_=1.505,w=0.466)

In this process, it is found that the optimal value of the inertia weight *w* is 0.466 rather than the empirical value of 0.5. Also, the best value of the learning factor is *c*_1_ = 2.99 and *c*_2_ = 1.505 rather than the empirical value and *c*_2_ = 2. Thus, it shows that the MOFOPSO algorithm designed in this paper achieves good convergence and efficiency when it is applied to solve the IK of the 6-DOF manipulator.

### Convergence Rate Analysis

**Figure 9:**
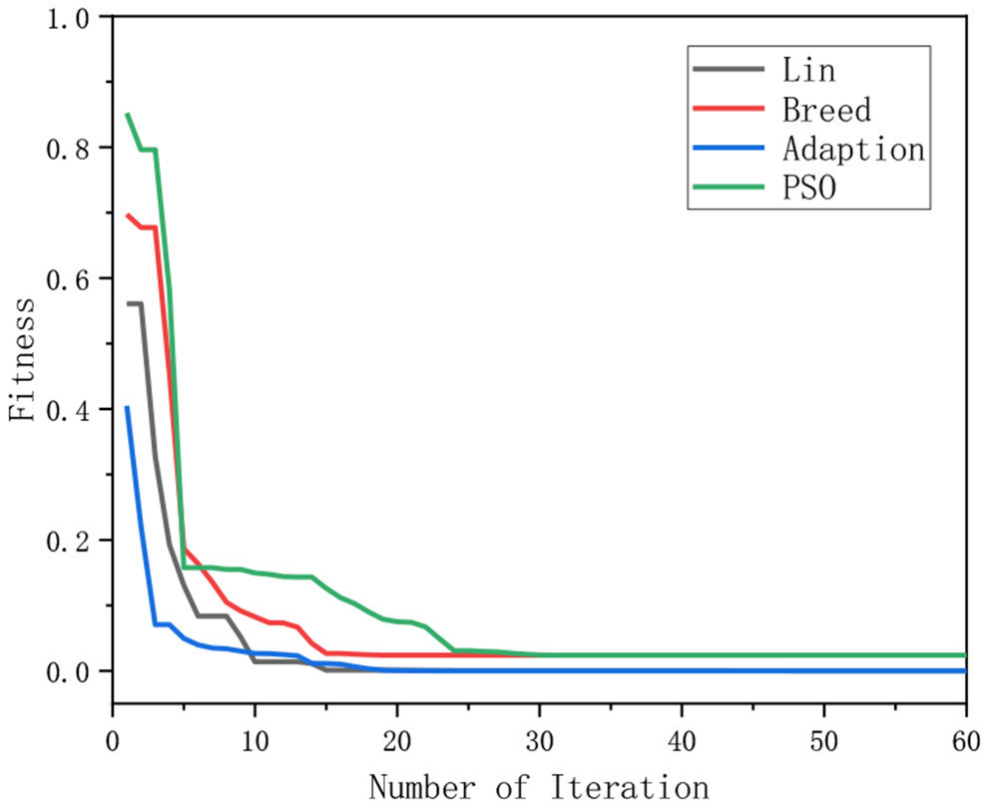
The relation of the fitness value and the number of iterations.

**Figure 10:**
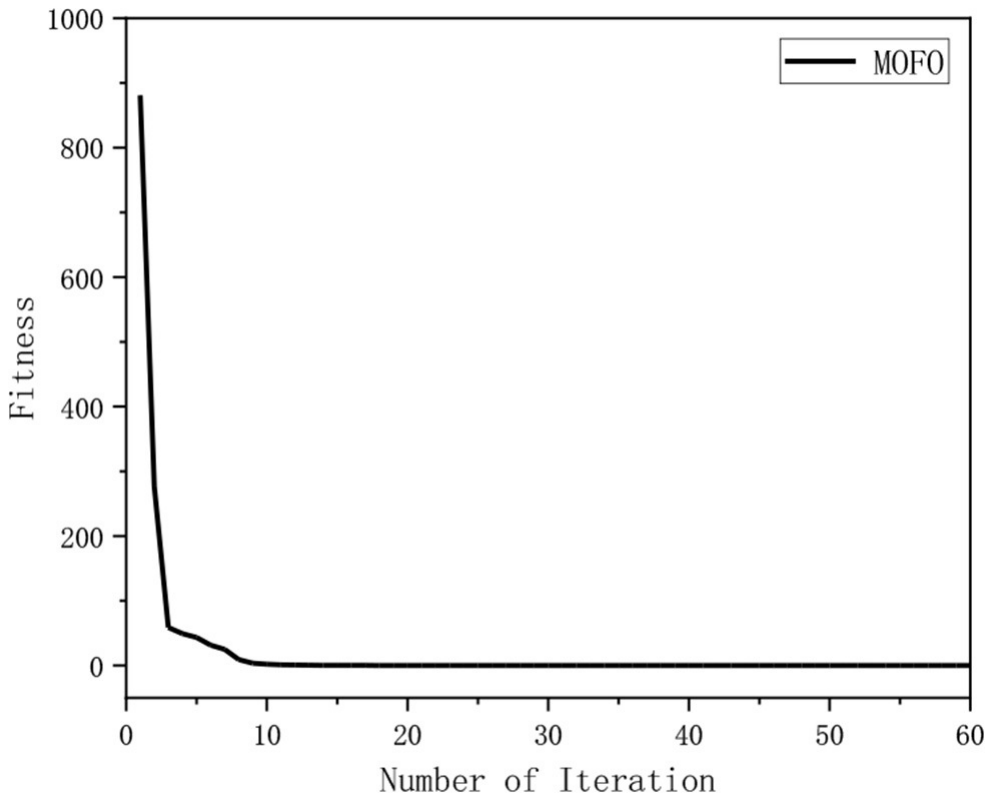
The relation between the fitness value and the number of iterations of the MOFOPSO algorithm.

In the simulation, the relationship between the fitness and the number of iterations for solving the IK of the 6-DOF manipulator using different algorithms is illustrated in Figure 9. It indicates that the MOFOPSO algorithm converges very fast and finds the optimal solution successfully. Due to the large difference in the order of the fitness function value of different algorithms, the convergence speed of the MOFOPSO algorithm is not obvious. Thus, the simulation results of the MOFOPSO algorithm are extracted separately, which are shown in Figure 10. It shows that the MOFOPSO reaches convergence after about nine iterations, indicating a very fast convergence speed of the MOFOPSO algorithm.

It should be noted that the initial value (in Figures 9, 10) is the fitness function value, the relation between the fitness value and the number of iterations of these algorithms can indicate the convergence speed of the algorithm (Serkan, 2021).

### Stability Analysis

**Figure 11:**
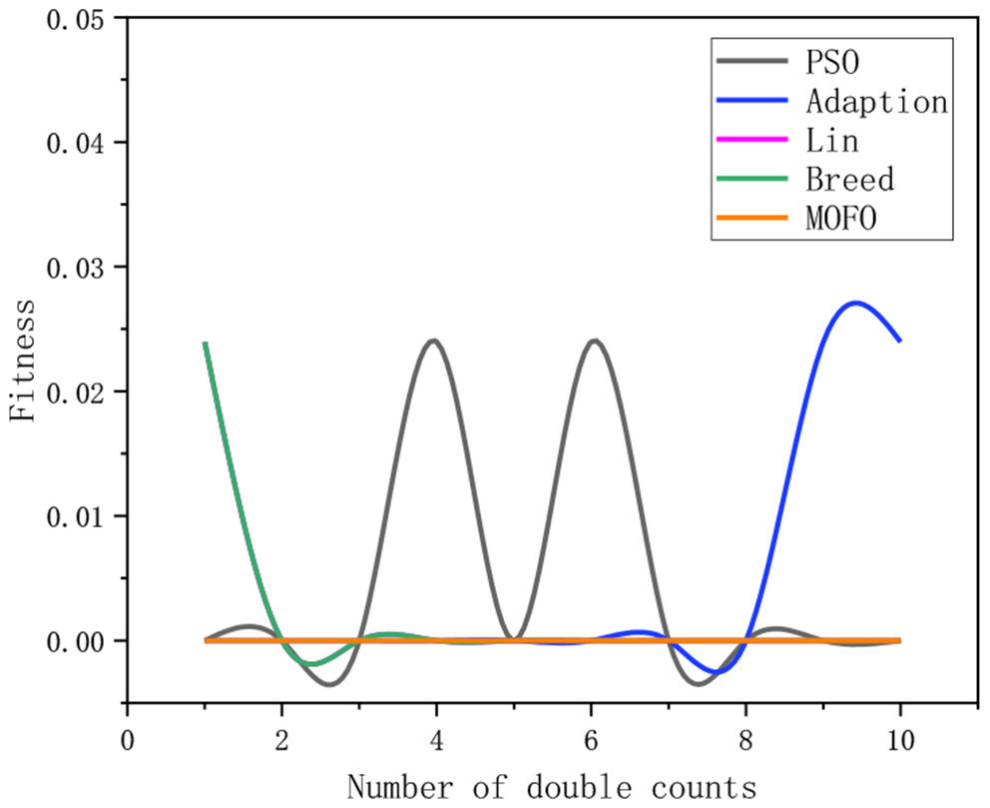
The stability of different algorithms.

It is acknowledged that stability is a prerequisite for the operation of an algorithm. If there is no guarantee of stability, it is meaningless to discuss the convergence of the algorithm. Therefore, this paper conducts experiments on the stability of the algorithms. Figure 11 shows the average value of the fitness function obtained by applying the algorithms to solve the IK of the 6-DOF (each algorithm is run 10 times consecutively). It indicates that the average fitness value of the classical PSO algorithm and RWPSO algorithm fluctuates greatly, indicating the poor stability of these two algorithms. In contrast, the average fitness value of the MOFOPSO algorithm is basically unchanged, indicating that the algorithm has good stability.

### Synthetic Simulation Results Analysis

Table 3 lists the comprehensive results of solving the IK of the manipulator using seven different algorithms including MOFOPSO. By analyzing the data in Table 5, the PSO algorithms based on natural selection and simulated annealing obtain the worst accuracy and large minimum values of the fitness function. The precision of the classical PSO algorithm, RWPSO algorithm, LDPSO algorithm, and hybridization-based PSO is all better. The MOFOPSO algorithm proposed in this paper achieves the highest precision, and it improves the fitness value to six orders of magnitude compared with other algorithms. In terms of solving speed, the proposed MOFOPSO algorithm is the fastest, about 0.7 s faster than the fastest one among other algorithms with the same accuracy, and the speed is increased by about 20%.

**Table 5:** The simulation results of different algorithms.

**PSO variants**	**Parameters**	**Average fitness**	**Average time**
PSO	c_1_=2,c_2_=2,w=0.5	0.004791622	3.3710435
PSO_Adaptation	c_1_=2,c_2_=2,w_max_=0.8,w_min_=0.2	0.004791622	5.0460895
PSO_Breed	c_1_=2,c_2_=2,w=0.5,b_c_=0.8,b_s_=0.05	0.002395811	3.8617253
PSO_Lamda	c_1_=2,c_2_=2,lamda=0.99	8.605051908	3.0658353
PSO_Lin	c_1_=2,c_2_=2,w_max_=0.8,w_min_=0.2	0.002395811	4.9974822
PSO_Nature	c_1_=2,c_2_=2,w=0.5	10.31912828	1.6046351
MOFOPSO	C_max_=3,c_min_=1.5,w_max_=0.8,w_min_=0.2 (c_1_=2.99,c_2_=1.505,w=0.466)	4.39E-09	2.6588259

In summary, Table 6 shows the angle values of the six joints of the manipulator calculated by different algorithms. The standard value is the joint Angle value of the manipulator obtained by mathematical derivation. It can be seen from the calculation results that the full-parameter optimization multi-objective particle swarm optimization algorithm proposed in this paper has the highest accuracy.

**Table 6:** The angle values of the six joints of the manipulator calculated by different algorithms.

**Algorithm**	**1-DOF**	**2-DOF**	**3-DOF**	**4-DOF**	**5-DOF**	**6-DOF**
Standard value	−1.047198	−1.047198	1.047198	−0.785398	0.785398	0.523599
PSO	−0.49164228	−0.9547942	0.003137195	−1.42360713	1.275797884	1.306460089
PSO_Adaptation	−1.1199608	−1.58311273	0.63145572	−0.16697007	1.031499907	−0.33419752
PSO_Breed	−1.08357914	−1.31515517	0.839326647	−0.47618412	1.132207126	0.185101802
PSO_Lamda	0.557517444	−0.12569131	−0.71358997	1.027890172	−2.00106961	2.265754194
PSO_Lin	−1.71189772	−1.31515511	0.839326614	−0.47618413	0.673626419	0.643682534
PSO_Nature	−2.54977472	0	0	0	0	0
MOFOPSO	−1.04719681	−1.04719668	1.047197423	−0.78539912	0.785401078	0.523591577

## Conclusion

In this paper, the problem of solving the inverse kinematics of the manipulator is analyzed firstly, and the advantages and disadvantages of the existing solution algorithms are summarized. Then, the PSO algorithm based on the principle of multi-objective optimization is selected to solve the IK of the manipulator. Next, the MOFOPSO algorithm is proposed and realized. Finally, to obtain a better inverse kinematics solution of the 6-DOF manipulator,this paper tests the performance of MOFOPSO with three typical functions. Also, six algorithms are taken for performance comparison. The experimental results indicate that the proposed method achieves good generalization ability, convergence speed, and accuracy.

## Data Availability Statement

The original contributions presented in the study are included in the article/supplementary material, further inquiries can be directed to the corresponding authors.

## Author Contributions

DL planned the study design. YH collected the data. SL and DC conducted experiments, analyzed the data, and wrote the paper. All authors contributed to the article and approved the submitted version.

## Funding

This work was supported by the Taishan Scholar Project of Shandong Province (ts20190937) and Universities Serving Qingdao Industrial Development Science and Technology Project.

## Conflict of Interest

The authors declare that the research was conducted in the absence of any commercial or financial relationships that could be construed as a potential conflict of interest.

## Publisher's Note

All claims expressed in this article are solely those of the authors and do not necessarily represent those of their affiliated organizations, or those of the publisher, the editors and the reviewers. Any product that may be evaluated in this article, or claim that may be made by its manufacturer, is not guaranteed or endorsed by the publisher.
